# Clinical and diagnostic features of angiosarcoma with pulmonary metastases: A retrospective observational study

**DOI:** 10.1097/MD.0000000000008033

**Published:** 2017-09-08

**Authors:** Hanping Wang, Juhong Shi, Hongrui Liu, Yeye Chen, Yining Wang, Wenze Wang, Li Zhang

**Affiliations:** a Department of Respiratory Medicine; b Pathology Department; c Department of Thoracic Surgery; d Radiology Department, Peking Union Medical College Hospital, Dong Cheng District, Beijing, China.

**Keywords:** angiosarcoma, cardiac angiosarcoma, diffuse alveolar hemorrhage, hemoptysis, metastasis

## Abstract

Angiosarcoma with pulmonary metastasis is a rare, fatal disease that often presents with multiple pulmonary nodules and diffuse alveolar hemorrhage. We herein review the detailed clinical characteristics of pulmonary metastatic angiosarcoma and determine a reasonable diagnostic strategy.

The medical records of 11 patients with pulmonary angiosarcoma were reviewed.

The mean age of the patients was 45.7 years (range, 30–71 years). All patients were male. The most common symptom was hemoptysis (8/11). Common initial misdiagnoses were tuberculosis (5/11), vasculitis (2/11), nontuberculous infectious disease (1/11), and constrictive pericarditis (1/11). Chest computed tomography (CT) of patients with hemoptysis showed bilateral, randomly distributed, variably shaped, and differently sized nodules, as well as ground-glass opacities (GGO) (8/11). The right heart was the most common primary tumor site (8/11), but the sensitivity of echocardiography was limited; CT angiography and cardiac magnetic resonance imaging (MRI) revealed more atrial masses. CT-guided needle biopsy was difficult to perform in most patients because of the small size of the nodules. The diagnosis was made by surgical biopsy of either the lung (3/9) or heart (6/9). The median overall survival of patients who underwent lung biopsy and those who underwent cardiac/pericardiac biopsy was 4.1 and 1.4 months, respectively (*P* = .098). The median overall survival of the 9 available patients was 5.0 months (95% confidence interval, 0.500–8.544).

Angiosarcoma with pulmonary metastases should be considered in patients with hemoptysis and concurrent GGO and nodules on their chest CT scan. Careful cardiologic monitoring is necessary for these patients, even without any cardiac symptoms or signs, and enhanced cardiac MRI is the first recommendation. Surgical biopsy is reliable for histological diagnosis, but the safety of the lung biopsy should be carefully assessed. When primary cardiac tumors are identified, heart biopsy should be preferentially considered.

## 1 Introduction

Angiosarcoma is a rare, fatal, malignant vascular tumor that accounts for <2% of all sarcomas.^[1]^ Chemotherapy is ineffective, and the prognosis is poor.^[2]^ The most common locations for the primary tumor are the skin, heart, liver, spleen, breast, bone, and gastrointestinal tract.^[3–5]^ Metastasis occurs in more than half of all patients, and the lung is the primary organ involved.^[2,3,6,7]^ Patients with pulmonary metastases often present with respiratory-associated symptoms such as hemoptysis, cough, and dyspnea. Patients are often initially misdiagnosed because of the nonspecific clinical manifestation and low clinical suspicion for the disease, which often leads to late diagnosis and a poorer prognosis. Some patients fail to be diagnosed while they are alive^[2,7,8]^; in fact, most patients are diagnosed at autopsy.^[6,9]^ Several case reports and small series have described patients with angiosarcoma in the lung (primary or metastatic).^[6,10,11]^ Most of these reports were written by pathologists, and they primarily focused on the histological characteristics of the disease, thereby providing good criteria for the histopathological diagnosis.^[7,11]^ The largest series highlighting the clinical manifestations of this condition was reported by Patel and Ryu^[11]^ from the Mayo Clinic. However, more reports are needed to identify detailed clinical clues for diagnosis. We herein analyzed 11 patients with angiosarcoma who were hospitalized at our institute from 2005 to 2013. We focused on the clinical characteristics of angiosarcoma and attempted to characterize this rare disease based on its clinical course and diagnostic clues.

## 2 Methods

### 2.1 Patients

**Figure 1:**
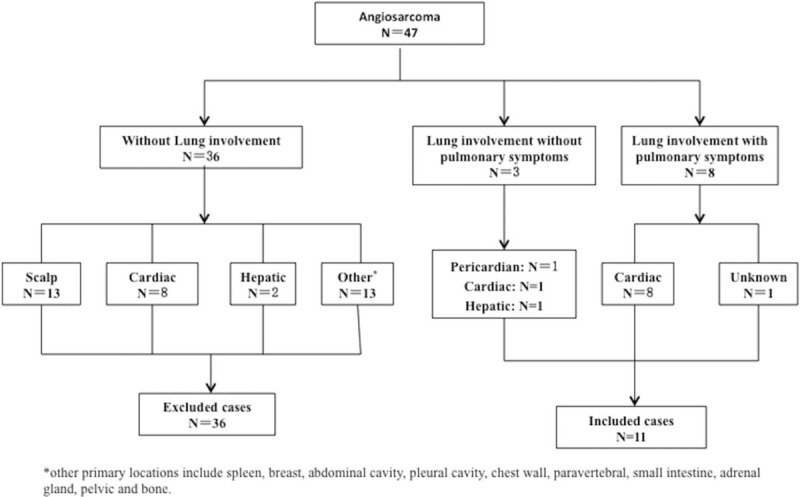
Flow diagram of the patient recruitment process.

The clinical data of 47 patients who were definitively diagnosed with angiosarcoma from January 2001 to January 2016 at Peking Union Medical College Hospital were extracted from the hospital database. The locations of the primary tumor included the heart, pericardium, scalp, liver, pelvic cavity, bone, chest wall, and small intestine. Among these 47 patients, 11 were found to have pulmonary involvement and were included in this retrospective study. Patients without pulmonary involvement were excluded. The details of the recruitment process are shown in Fig. 1.

This study was approved by the Peking Union Medical College Hospital Institutional Review Board.

### 2.2 Diagnosis

Definitive diagnoses were based on a review of the biopsy-based histological findings and were confirmed by a senior pathologist. Immunohistochemical staining for CD31, CD34, and factor VIII-related antigen was performed to determine the tumor origin; specimens were confirmed as vascular tumors originating from endothelial cells when at least one of these antigens was present.

### 2.3 Clinical and radiologic features

Details regarding each patient's history, including symptoms, diagnostic work-up, and initial misdiagnosis (if applicable), were obtained from the medical records. The radiologic findings were based on a review of the relevant radiologic studies and confirmed by a senior radiologist. The search for and identification of the location of the primary tumor were described. The biopsy sites and operation methods were reviewed, as was the outcome of the biopsy procedure (changes in symptoms and morbidity during the perioperative period). Treatment and response were also retrospectively evaluated. The survival outcomes were obtained from the medical records or by telephone follow-up.

### 2.4 Statistical analysis

Statistical analyses were performed using IBM SPSS Statistics for Windows (Version 19.0; IBM Corp., Armonk, NY). Categorical variables are presented as frequencies with percentages. Overall survival was estimated using the Kaplan–Meier method.

## 3 Results

### 3.1 General characteristics

Of the 47 medical records of patients with angiosarcoma extracted from the hospital database, 11 male patients fulfilled the criteria for angiosarcoma with pulmonary involvement. The clinical findings are detailed in Table 1. The patients’ mean age was 45.7 years (range, 30–71 years). Of these 11 patients, 8 were heavy smokers. No other special exposure history was identified.

**Table 1:**
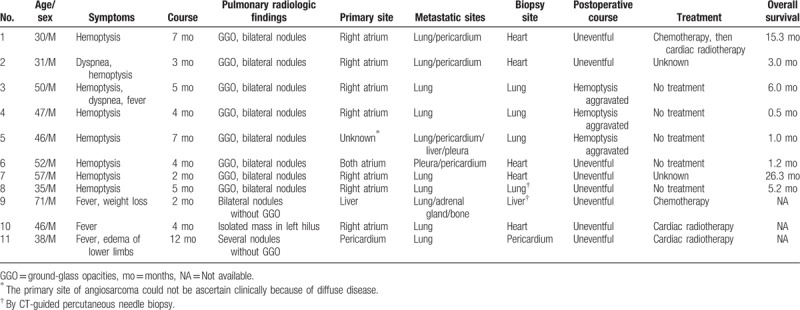
Clinical findings in 11 patients with pulmonary metastatic angiosarcoma.

### 3.2 Symptoms

Pulmonary symptoms were the chief presenting complaint in 8 patients, while pulmonary involvement was identified during screening and obvious respiratory-associated symptoms were absent in the remaining 3 patients. The mean interval from symptom onset to diagnosis was 5.0 months (range, 2–12 months). Gradually increasing hemoptysis (8/11, 72.7%) was the most common initial and presenting symptom. The other symptoms included shortness of breath (5/11, 45.5%), cough (3/11, 27.3%), pleuritic chest pain (3/11, 27.3%), and intermittent fever (5/11, 45.5%). Fever was the main presenting symptom in the 3 patients without pulmonary symptoms. The patients also reported nonspecific symptoms including night sweats, fatigue, and weight loss. Symptoms related to the location of the primary tumor included dyspnea when lying down, edema of the lower extremities, and oliguria secondary to heart failure (4/11, 36.4%). The most common misdiagnoses before the final diagnosis included tuberculosis (5/11, 45.5%), vasculitis (2/11, 18.2%), nontuberculous infectious disease (1/11, 9.1%), and constrictive pericarditis (1/11, 9.1%). No special laboratory abnormalities were found in these patients except for mild anemia (4/11, 36.4%) or mild hypoxemia.

### 3.3 Radiological presentation, primary site, and metastatic sites outside the lung

**Figure 2:**
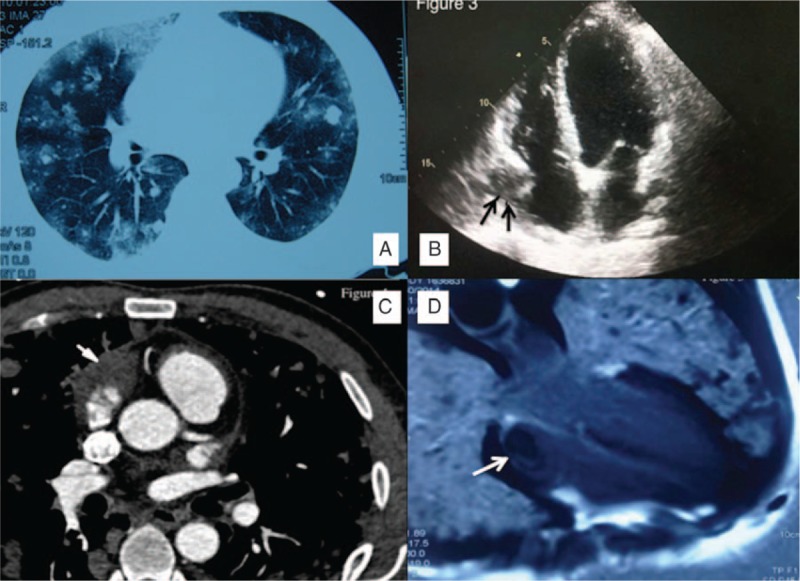
Radiological presentations of pulmonary metastatic angiosarcoma. (A) Chest computed tomography of pulmonary metastatic angiosarcoma. Multiple unequally sized and sharp-margined nodules are seen, mostly distributed in the peripheral regions. Some ground glass opacities are seen, either surrounding the nodules or unassociated with nodules. (B) Echocardiographic manifestation of cardiac angiosarcoma. A mass is seen arising from the lateral wall of the right atrium (black arrows). (C) Appearance of right atrial angiosarcoma on CT angiography. An irregular soft tissue mass is seen in the right atrium (white arrow). (D) Right atrial angiosarcoma on enhanced cardiac MRI. A low signal was found in the right atrium, but the echocardiographic study was negative (white arrow).

Chest computed tomography (CT) of 8 patients with hemoptysis showed bilateral, randomly distributed, variably shaped, and differently sized nodules surrounded by ground-glass opacities (GGO) (8/11, 72.7%); the nodules ranged in size from 0.5 to 2.0 cm, and patchy GGO without nodules were also noted (Fig. 2A). CT of patients without pulmonary symptoms showed an isolated mass in the left hilus (1/11, 9.1%) and several nodules in the lung without GGO (2/11, 18.2%). Pleural effusion (4/11, 36.4%) and pericardial effusion (5/11, 45.5%) were also observed. Positron emission tomographic scans were performed in 4 patients; 2 showed multiple nodules with high fluorodeoxyglucose uptake, and 1 showed masses with high fluorodeoxyglucose uptake in both the left hilus and right atrium, indicating malignant disease.

**Figure 3:**
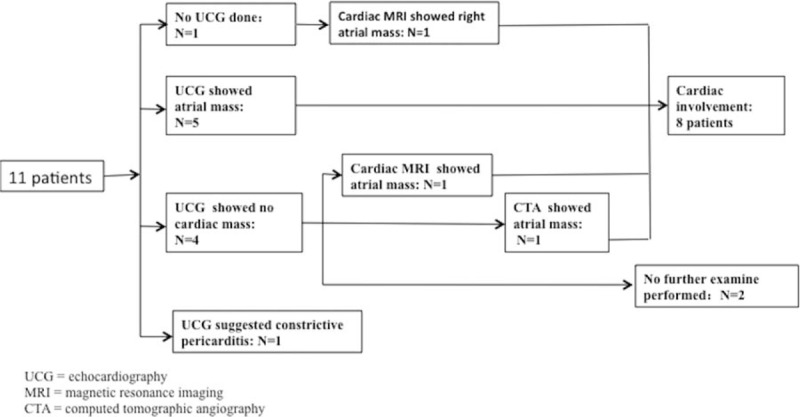
Cardiologic evaluations of 11 patients. Echocardiography was conducted in 10 patients, and atrial masses were found in 5 of them. In the one patient who did not undergo echocardiography, an atrial mass was observed by cardiac magnetic resonance imaging. Computed tomography angiography and cardiac magnetic resonance imaging revealed an atrial mass in 2 of the 3 patients with negative echocardiographic results.

The cardiologic evaluation findings are summarized in Fig. 3. Because most of the nodules shown on high-resolution CT exhibited a random distribution indicative of angiogenic disease, the heart was carefully evaluated in almost all patients. Echocardiography was conducted in 10 patients, and atrial tumors were identified in half (5/10) of them (Fig. 2B); constrictive pericarditis was suggested in 1 patient. Of the remaining 4 patients, CT angiography (Fig. 2C) and cardiac MRI (Fig. 2D) revealed atrial masses in 2 patients. In the only patient who did not undergo echocardiography, an atrial mass was observed by cardiac MRI. (The patient had severe hemoptysis and a cardiac murmur; thus, cardiac MRI was selected instead of echocardiography.)

The primary site was the atrium (right atrium or both) in 8 patients, pericardium in 1, and liver in 1; the remaining patient showed diffuse disease involving the lung, pericardium, pleura, and liver (multiple nodules) by positron emission tomography–CT, but we failed to identify the primary location. The pericardium was the second most common metastatic site after the lung.

### 3.4 Tissue biopsy

All diagnoses were made by tissue biopsy. The biopsy sites are listed in Table 1.

Bronchoscopy was conducted in six patients (all had hemoptysis). The main finding was fresh blood in the airways. No endobronchial lesion was observed after blood removal or bronchial washing. All bronchoalveolar lavage fluid samples had a uniformly bloody appearance, suggesting alveolar hemorrhage. Microscopic examination revealed a large number of erythrocytes, but no specific changes such as malignant cells were present. Transbronchial lung biopsy was performed in one patient, but no evidence of malignancy was found.

CT-guided percutaneous transthoracic and transhepatic needle biopsies were each performed in one patient, respectively, yielding a positive diagnosis in both.

Nine patients were diagnosed by surgical biopsy, 5 by cardiac mass biopsy, 3 by lung biopsy using video-assisted thoracic surgery, and one by pericardiectomy. Among the 5 patients who underwent cardiac mass biopsy, the lesions were observed on the right atrial wall in all 5 patients, and pericardial involvement (mass or pericardial effusion) was observed in 3 of the 5 patients. Among the patients who underwent cardiac/pericardiac biopsy, no serious postoperative events were observed. Heart failure improved postoperatively after draining the pericardial effusion and resecting the mass. No deaths occurred during the postoperative period. All patients who underwent lung biopsy developed postbiopsy exacerbation of pulmonary hemorrhage and progressive shortness of breath; 1 patient died of severe pulmonary hemorrhage 10 days after the surgical biopsy. The median overall survival of the patients who underwent lung biopsy and cardiac/pericardiac biopsy was 4.1 and 1.4 months, respectively (*P* = .098).

### 3.5 Pathology

**Figure 4:**
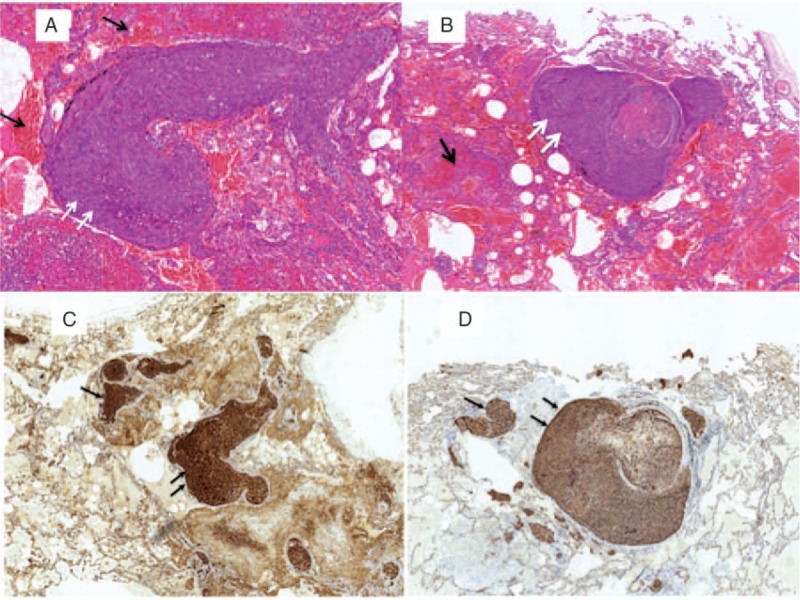
Lung biopsy showing: (A and B) Multiple hemorrhagic (black arrows) and vasoformative lesions made up of atypical spindle tumor cells (white arrows) (HE stain). (C and D) Immunohistochemical staining for the endothelial markers CD31 and CD34 were positive, demonstrating the endothelial origin of the neoplastic cells.

The primary histological characteristics were regions of solid growth lined by spindle tumor cells on hematoxylin/eosin staining with fresh hemorrhage within tumor nodules and in the immediately adjacent lung parenchyma (Fig. 4A and B). The immunohistochemical features were at least 1 positive vascular marker, including CD31 (Fig. 4C), CD34 (Fig. 4D), and Von Willebrand factor. The staining for AE1/AE3 was often negative.

### 3.6 Treatment, survival, and prognosis

Among the patients with pulmonary symptoms, only 1 patient underwent antitumor therapy (paclitaxel chemotherapy weekly for 2 cycles). Bone metastasis and lung lesion enlargement were then noted, and radiotherapy was administered to the heart. The patient's overall survival was 15.3 months. The remaining patients only received supportive therapy because of their poor physical status. The median overall survival of the 8 patients was 3.0 months (95% confidence interval, 0.5–8.5).

Among patients without pulmonary symptoms, cardiac radiotherapy was conducted in patients with pericardial angiosarcoma and atrial angiosarcoma; the last patient with liver angiosarcoma underwent one cycle of chemotherapy. Their survival data were unknown after they were discharged.

## 4 Discussion

Angiosarcoma with pulmonary metastasis tends to occur in young males. The reported morbidity ratio in males and females is 2:1 to 3:1, respectively,^[11]^ but the actual ratio may be greater. The median duration between the onset of symptomatic disease and diagnosis in the present series was 5 months (range, 2–12 months), highlighting the rapid progression of the disease.

Angiosarcoma with pulmonary metastasis is often misdiagnosed because of its rare occurrence and nonspecific symptoms. Most angiosarcomas are thought to originate from the vascular endothelium and spread through the bloodstream to the lung, eventually eroding and obstructing the pulmonary vessels. The result is high pressure in the pulmonary circulation and the destruction of small pulmonary vessels. Therefore, either diffuse alveolar hemorrhage (DAH) or pulmonary embolism (presenting as chest pain, hypoxemia, and hemoptysis) is often the presenting manifestation.^[6,12]^ Hemoptysis, which can mimic the symptoms of pulmonary hemorrhage syndrome, pulmonary vasculitis, pulmonary infection, or parenchymal lung disease, is the most commonly reported symptom.^[6,11,13]^ Antibiotics and steroid therapy can sometimes improve the symptoms.^[14]^ In the present study, 4 patients experienced remission of hemoptysis with antibiotics and steroid therapy. We hypothesize that steroids cause a temporary lessening of the vascular inflammatory response, leading to a decrease in vascular permeability. This remission might result in the misdiagnosis of pulmonary vasculitis.^[6]^ Infection is yet another common misdiagnosis, particularly tuberculosis in China or India, where the morbidity of tuberculosis is relatively high.^[8]^ For patients with concurrent hemoptysis and cardiac dysfunction, Behçet disease is also a potential misdiagnosis. Misdiagnosis often delays the actual diagnosis, which results in a higher risk of biopsy and lower chance of oncologic treatment; some patients have even lost the chance for diagnosis while still alive.

The radiological manifestations of pulmonary metastatic angiosarcoma are characteristic: concurrent GGO and multiple compact nodules with sharp borders in the lung. In the present study, the nodules were usually of different sizes and tended to be distributed more in the peripheral region than in the central region. This finding aligns with the findings in many previous case reports and small series.^[6,7,9]^ The distinctive appearance of some angiosarcomatous pulmonary metastases, such as thin-walled cysts, has been reported in association with origination of the tumor in the scalp. Spontaneous hemopneumothorax has also been reported.^[15]^

Despite its rarity, cardiac angiosarcoma accounts for one-third of malignant cardiac tumors.^[16,17]^ It is also the most common primary tumor from which pulmonary metastases originate.^[6,7,11]^ The right atrium is the most common site for cardiac angiosarcomas; however, clinicians generally have a low level of clinical suspicion for this disease because of the absence of specific symptoms, and metastasis is present in 66% to 89% of patients at the time of diagnosis.^[18]^ A careful cardiac examination is needed in patients with multiple pulmonary nodules and DAH. Echocardiography has been most widely performed, but its sensitivity for atrial masses is unsatisfactory.^[16,19,20]^ Its sensitivity in our series was 75%. This suggests that other methods are needed to thoroughly exclude cardiac masses in patients with negative echocardiography. MRI and transesophageal echocardiography could be more promising because of their higher resolution and sensitivity.^[8,21]^ MRI is useful in detecting the tumor and depicting its contour and relationship with other cardiac structures.^[21]^ MRI with gadolinium enhancement could provide more useful information for the differential diagnosis.^[22]^ Therefore, MRI is an essential tool for detection and preoperative evaluation of cardiac angiosarcoma.^[21]^

The definitive diagnosis of angiosarcoma is solely based on histopathologic examination of biopsy tissue.^[16]^ However, the biopsy process is often difficult. Due to the rapid progression of the disease, many patients do not have the opportunity to undergo biopsy. Transbronchial lung biopsy is the safest method, but its role is limited in detecting diffusely metastatic angiosarcoma in the lung.^[6]^ Transthoracic needle biopsy and transbronchial biopsy fail to provide diagnostic evidence of angiosarcoma. Pulmonary hemorrhage in the lung often overshadows other histologic findings related to angiosarcoma,^[6]^ and the finding of alveolar hemorrhage without an identifiable tumor is sometimes considered to suggest nonneoplastic DAH syndrome.^[6,19,23]^

Surgical biopsy is a reliable method for diagnosis in selected patients.^[7]^ Biopsy can be performed either on the primary or on a metastatic tumor, such as in the lung. Our study showed that the safety of the procedures differed based on the postoperative course. Because of the high pulmonary arterial pressure in affected patients, an operative incision would worsen the bleeding, which could lead to delayed healing of the incision and even death. According to prior reports, several patients died in the 4-week postoperative period after lung biopsy.^[6,19]^ However, in patients undergoing cardiac operations in our series, the cardiac functions improved after the atrial tumor was removed and the pericardiac effusion drained. Furthermore, several special methods, such as cardiopulmonary bypass and retrograde cannulation of the inferior vena cava, could be used to increase the safety of cardiac operations.^[16]^ We suggest that surgical lung biopsy should be carefully performed with consideration of perioperative safety. When a cardiac mass has been identified, cardiac biopsy should be considered before lung resection.

The main limitation of this study is the small number of patients, primarily because of the rarity of the disease. More patients and additional details are needed to investigate this rare but fatal condition. Clinicians must be aware of this condition when a patient's symptoms and CT findings suggest this entity so that diagnosis can be achieved in a timely manner and proper treatment can be started.

In conclusion, angiosarcoma with pulmonary metastases is a rare disease that should be considered in patients with hemoptysis and concurrent GGO and nodules on their chest CT scan. Careful cardiologic monitoring is necessary for these patients, even without any cardiac symptoms or signs, and enhanced cardiac MRI is the first recommendation. Surgical biopsy is reliable for a histological diagnosis, but the safety of the lung biopsy should be carefully assessed. When primary cardiac tumors are identified, a heart biopsy should be preferentially considered.
